# Pre-Exercise Hyperpnea Attenuates Exercise-Induced Bronchoconstriction Without Affecting Performance

**DOI:** 10.1371/journal.pone.0167318

**Published:** 2016-11-29

**Authors:** Philipp A. Eichenberger, Thomas A. Scherer, Christina M. Spengler

**Affiliations:** 1 Exercise Physiology Lab, Institute of Human Movement Sciences and Sport, ETH Zurich, Zurich, Switzerland; 2 LungenZentrum Hirslanden, Zurich, Switzerland; 3 Zurich Center for Integrative Human Physiology (ZIHP), University of Zurich, Zurich, Switzerland; National and Kapodistrian University of Athens, GREECE

## Abstract

Whole-body warm-up exercises were shown to attenuate exercise-induced bronchoconstriction (EIB). Whether intense pre-exercise hyperpnea offers similar protection and whether this might negatively affect exercise performance is unknown. Nine subjects with EIB (25±5 yrs; forced expiratory volume in 1s [FEV_1_], 104±15% predicted) performed an exercise challenge (ECh) followed—after 30min—by a constant-load cycling test to exhaustion. The ECh was preceded by one of four conditions: by i) control warm-up (CON) or by 10min of normocapnic hyperpnea with partial rebreathing at either ii) 50% (WU50) or iii) variable intensity (8x 30s-80%/45s-30%; WU80/30), or at iv) 70% (WU70) of maximal voluntary ventilation. FEV_1_ was measured at baseline and in 5-min intervals until 15min after CON/warm-up and 30min after ECh. None of the warm-up conditions induced EIB. The maximal post-ECh decrease in FEV_1_ was -13.8±3.1% after CON, −9.3±5.0% after WU50 (p = 0.081 vs. CON), −8.6±7.5% after WU80/30 (p = 0.081 vs. CON) and −7.2±5.0% after WU70 (p = 0.006 vs. CON), and perception of respiratory exertion was significantly attenuated (all p≤0.048), with no difference between warm-up conditions. Only after CON, FEV_1_ remained significantly reduced up to the start of the cycling endurance test (−8.0±4.3%, p = 0.004). Cycling performance did not differ significantly between test days (CON: 13±7min; WU50: 14±9min; WU80/30: 13±9min; WU70: 14±7min; p = 0.582). These data indicate that intense hyperpnea warm-up is effective in attenuating EIB severity and accelerating lung function recovery while none of the warm-up condition do compromise cycling performance.

## Introduction

Regular physical exercise is increasingly recognized to improve not only cardio-pulmonary functioning but also asthma-specific pathophysiological changes like airway inflammation and hyperresponsiveness in asthmatics [1,2]. Nonetheless, most asthmatics show transient airway obstruction during and after strenuous exercise [3], commonly termed exercise-induced bronchoconstriction (EIB). Interestingly, improved EIB was observed in asthmatics in a second of two EIB-inducing exercise trials that were ≤4 hours apart [4,5]. This so-called refractory effect, i.e. a period of activity reducing the extent of EIB during a subsequent period of activity, was also observed when the EIB-inducing exercise was preceded by exercise with a different protocol (usually termed “warm-up”) in most [6–11] but not all studies [12], as recently summarized by Stickland et al. [13] in a systematic review. Similarly, decreased EIB after exercise was shown when 6min of intense hyperpnea (~78% maximal voluntary ventilation, MVV) were performed 30-50min before the physical exercise challenge [14]. The refractory effect induced by non-pharmacological means is of particular interest for athletes since preventive regular intake of anti-EIB medications (β_2_‐agonists) could lead to EIB-worsening [15,16] and thus possibly limit effective participation in exercise in the long run.

However, whole-body warm-up as well as hyperpnea warm-up exercises bear the risk of inducing EIB by itself [6,7,9,11,14] which might compromise preparation for the subsequent competition. Interestingly, one study showed that exercising with warm and humid air did not induce substantial EIB but this exercise was still effective in preventing EIB in the following exercise challenge [17]. From a practical point of view, however, warm-up exercise using warm and humid air is a technically challenging approach. An alternative approach might be volitional, isolated hyperpnea with partial rebreathing, keeping the inspirate warm and humid. However, it remains unknown whether hyperpnea with warm and humid air, likely not inducing bronchoconstriction [18], would be equally effective in reducing EIB as was shown for hyperpnea with dry air that was used previously [14], inducing bronchoconstriction by itself. Furthermore, it is unclear which hyperpnea protocol would provide the best protection since different exercise warm-up protocols were shown to reduce EIB to different degrees [13]. For whole-body exercise warm-up, Stickland et al. [13] concluded in their systematic review that at least some high intensity exercise is needed to reduce EIB in a following exercise. Because ventilation is frequently not reported in warm-up exercise trials and because systemic effects of muscle activity are different between whole-body exercise and volitional breathing, it is difficult to determine the optimal intensity of volitional breathing required for similar warm-up effects. In this context, also a potential side-effect needs consideration, i.e. hyperpnea intensities ≥70% MVV were shown to induce respiratory muscle fatigue lasting up to 60min into recovery [19]. This might, in fact, be disadvantageous for subsequent exercise performance where respiratory muscle fatigue may further develop and compromise performance [20].

Therefore, the aim of the present study was to assess the effect of different pre-exercise hyperpnea intensities, i.e. moderate- and high-intensity continuous hyperpnea as well as interval-type hyperpnea with warm and humid air, on 1) inducing bronchoconstriction after hyperpnea, 2) attenuating EIB after a subsequent exercise challenge and 3) affecting exercise performance.

## Method

### Subjects

Nine subjects (6 females [one competitive swimmer, 14-16h/wk], 3 males; Table 1) with a history of EIB and a ≥10% decline in forced expiratory volume in 1s (FEV_1_) in a control exercise challenge (ECh) took part in this study. All subjects were non-smokers, were not taking any medication (apart from asthma medication, Table 1) and did not have any acute or chronic disease other than mild asthma (defined as controlled asthma with at most low-dose inhaled corticosteroids, i.e. budesonide ≤400μg d^-1^, [21]) and/or EIB or injuries that might affect performance. Participants gave their written informed consent to take part in this study. The study was approved by the ethics committee of the ETH Zurich and was performed according to the Declaration of Helsinki (2008 Revision).

**Table 1:** Characteristics of study participants.

Age (years)	24.8 ±4.5
Height (m)	1.71±0.10
Weight (kg)	69.3±8.7
BMI (kg·m^−2^)	23.7±1.5
**On controller medication (n = 4):**	
Inhaled corticosteroids (μg·d^−1^) ^§^	350±191
Long-acting β_2_-agonists (μg·d^−1^) ^§^	11±9
**On reliever medication only (n = 3)** **No medication (n = 2)**	
FVC (L)	5.03±0.84
FVC (% pred)	112±18
FEV_1_ (L)	3.99±0.75
FEV_1_ (% pred)	104±15
FEV_1_/FVC (%)	79.3±7.4
FEV_1_/FVC (% pred)	93±9
PEF (L·s^−1^)	8.35±1.50
PEF (% pred)	102±16
FEF_25-75%_ (L·s^−1^)	3.68±1.21
FEF_25-75%_ (% pred)	81±23
MVV (L·min^−1^)	143.2±28.0
MVV (% pred)	103±10
R5 (kPa·L^−1^·s^−1^)	0.30±0.06
R20 (kPa·L^−1^·s^−1^)	0.31±0.07
R5-R20 (kPa·L^−1^·s^−1^)	−0.01±0.01
X5 (kPa·L^−1^·s^−1^)	−0.10±0.02
AX (kPa·L^−1^)	0.20±0.08

Data are presented as mean ± SD (n = 9). BMI, body mass index; FVC, forced vital capacity; FEV_1_, forced expiratory volume in 1s; PEF, peak expiratory flow; FEF_25-75%_, forced expiratory flow between 25 and 75% FVC; MVV, maximal voluntary ventilation; R5, airway resistance at 5Hz; R20, airway resistance at 20Hz; R5-R20, difference in airway resistance measured at 5 and 20Hz; X5, airway reactance at 5Hz, AX, airway reactance area from 5Hz to resonance frequency; pred, predicted.

^§^ used in combination, all budesonide ≤400μg d^-1^.

### Study design

Subjects reported to the laboratory on six different days with at least 48h between each session. All test sessions were scheduled at the same time of day. Inhalation of long-acting and short-acting β_2_-agonists was discontinued for 48h and 8h, respectively, prior to each testing session (except for tests on the 1^st^ day) while subjects continued taking their maintenance medications, if appropriate, in analogy to a previous study [10]. Subjects completely refrained from physical exercise for 24h and from intake of caffeinated products on test days prior to testing.

**Fig 1:**
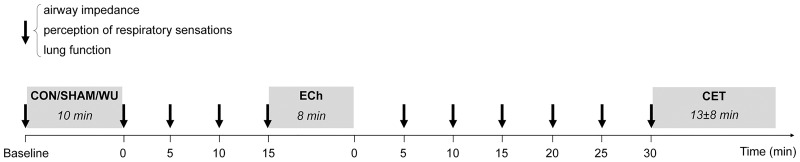
Study outline of 2^nd^ to 6^th^ visit. At baseline and in 5min intervals after control warm-up (CON), SHAM or hyperpnea warm-up (WU) and after the exercise challenge (ECh), airway impedance, perception of respiratory sensations and lung function (↓) were measured until 30min into recovery from ECh and the start of the constant-load cycling test to exhaustion (CET). Warm-up (WU) consisted of normocapnic hyperpnea at 10% (SHAM), 50% (WU50), variable intensity (8x 30s-80%/45s-30%; WU80/30) or 70% (WU70) maximal voluntary ventilation with tidal volume set at 50–60% of vital capacity. On 2^nd^ visit, all subjects performed CON followed by ECh and CET. On subsequent visits, subjects performed either SHAM, WU50, WU80/30 or WU70 in randomized and balanced order, followed by ECh and CET. See text for details.

In the 1^st^ visit, airway impedance, lung function and MVV were measured and subjects were familiarized with all procedures. In the following five visits (Fig 1), subjects performed either no warm-up (10min of rest = CON, always 2^nd^ visit), or one of four different 10-min hyperpnea warm-up exercises (including one sham warm-up) in a randomized and balanced order (3^rd^ to 6^th^ visit). Each of these 10-min pre-exercise interventions (2^nd^ to 6^th^ visit) was followed—after a 15-min break—by an 8-min ECh and—after a 30-min break—by a constant-load test (CET) to exhaustion. Prior to CON/warm-up and during the breaks, airway impedance, perception of respiratory sensations, and lung function were measured at 0, 5, 10 and 15min after CON/warm-up and at 2.5, 7.5, 12.5, 17.5, 22.5 and 27.5min after the ECh.

### Hyperpnea warm-up

Pre-exercise hyperpnea warm-up consisted of normocapnic hyperpnea performed for 10min with a device using partial rebreathing of expired air (SpiroTiger^®^, idiag, Fehraltorf, Switzerland) at a constant minute ventilation (${\dot{\text{V}}}_{\text{E}}$) corresponding to 10% (SHAM), 50% (WU50), or 70% MVV (WU70) or at alternating intensities of 80% and 30% MVV (8 x 30s at 80% interspaced by 45s at 30% MVV, [WU80/30]) with given respiratory frequency (f_R_) and tidal volume (V_T_; set at 50–60% forced vital capacity, FVC), reinforced by an experimenter, if necessary. End-tidal CO_2_ partial pressure (P_ET_CO_2_) was monitored using a metabolic cart (OxyconPro, Jaeger, Höchberg, Germany) to assure it stayed in the normocapnic range. In the CON condition, subjects rested in a chair for 10min.

### Exercise challenge and endurance test

The ECh consisted of cycling for 8min on a bicycle ergometer (Ergoline 800s / Ergoselect 200k, Ergoline, Blitz, Germany) while breathing dry air from a reservoir. Subjects wore a nose clip and breathed through a mouthpiece connected to the metabolic cart. Heart rate was recorded continuously using a heart rate monitor (s610i, Polar, Kempele, Finland). Progression of the workload was estimated [22] and then individually adjusted to reach a ${\dot{\text{V}}}_{\text{E}}$ ≥60% MVV by the 4^th^min, which was then kept constant for further 4min. Subjects chose their preferred pedaling frequency at the beginning of the tests which was then held constant throughout the study. The individual workload profiles for both, ECh and CET were determined in the CON condition and kept constant for all further testings. The CET protocol started with two submaximal 30-s stages (80% and 90% of the final ECh-workload), after which participants cycled at 100% of the final ECh-workload until volitional exhaustion. At baseline, every 2min during both exercise tests and at exhaustion (CET only) subjects rated their perception of respiratory sensations and leg exertion. After each rating, 20μl of capillary blood was drawn from an earlobe to analyse blood lactate concentration (BIOSEN C-line Sports, EKF-diagnostic, Barleben, Germany).

### Airway impedance

Airway resistance at 5Hz (R5) and 20Hz (R20) and the difference between the two (R5-R20), airway reactance at 5Hz (X5) and reactance area from 5Hz to resonance frequency (AX) were measured according to the current guideline [23] using the MasterScreen Impulse Oscillometry System (Jaeger, Hoechberg, Germany). At baseline, at least 3 measurements were performed and the average thereof was taken as the final value. In the breaks, one measurement per time-point was performed due to time restrictions.

### Lung function

Lung function was measured using the metabolic cart. FVC, FEV_1_, FEV_1_/FVC, peak expiratory flow (PEF), forced expiratory flow between 25 and 75% FVC (FEF_25−75%_) and MVV were measured according to current guidelines [24]. Predicted values were derived from Quanjer et al. [25,26].

### Respiratory sensations and leg exertion

Perception of breathlessness (the sensation of “not getting enough air”), respiratory exertion (“work/effort that is required by breathing”) and leg exertion (“work/effort that is required by cycling exercise”) were measured using a visual analogue scale with “no” as starting point and “maximal” as end point.

### Data analysis

A minimal sample size of nine was derived from published work by Dahlen et al. (2001) addressing reproducibility and sample size requirements of exercise-induced bronchoconstriction measurements [27]. Effects of pre-exercise hyperpnea and ECh on lung function and airway impedance were assessed by comparing values after warm-up and after ECh with values at baseline. Changes in CON and SHAM conditions did not significantly differ. Since three subjects showed a potential response to the SHAM intervention, effects of the different WU-strategies were compared to CON (for details, please see in chapter *Changes after the ECh* and S1–S4 Figs). The degree of bronchoprotection by pre-exercise hyperpnea was calculated by subtracting the maximal decrease in FEV_1_ after ECh in the respective condition from the maximal decrease in the CON condition, and expressed as percentage of the CON condition.

Data were tested for normality using the Shapiro-Wilk-test and compared between conditions and time-points using repeated measures ANOVA with Bonferroni-corrections. To further assess mechanistic aspects, i.e. whether differences in bronchoprotection seen in this study would translate into improvements in airway resistance and respiratory sensations or whether differences in FEV_1_ seen in this study would be large enough to affect CET-performance at all, we compared 1) airway resistance and respiratory sensations in the trial with best bronchoprotection with the CON trial and 2) performance in the trial with the best (least compromised) FEV_1_, with performance in the CON trial using paired t-tests or, in case of non-normal distributed data, Wilcoxon signed rank test. Pearson’s correlation or, for non-normal distributed data, Spearman’s correlation was used to assess the relationship between maximal changes in FEV_1_ and airway impedance parameters. SPSS Statistics 21 (IBM Company, New York, USA) was used for statistical analyses. Values are given as mean ± SD unless otherwise stated. For all statistical tests p≤0.05 was considered significant.

## Results

Baseline lung function and airway impedance were not significantly different between days (S1 Table). Maximal changes in FVC, FEV_1_, PEF, FEF_25-75%_, R20, X5 and AX after the 10-min pre-exercise intervention were not different between CON and the hyperpnea warm-up conditions except for R5 which was slightly higher after WU80/30 warm-up compared to CON (S2 Table). In none of the instances, decreases in FEV_1_ were ≥10% (range: −8.3 to +7.5%).

### Physiological variables during the ECh

Workload, ventilation, gas exchange, heart rate, blood lactate concentrations, perception of breathlessness, respiratory and leg exertion in the ECh did not significantly differ between conditions (Table 2). An overall effect of warm-up on heart rate and perception of respiratory exertion was observed but with no significant differences between conditions.

**Table 2:** Physiological variables during the 8-min exercise challenges.

		CON	WU50	WU80/30	WU70	p-value
Workload (W)	m±SD	199.7±46.8	199.6±45.7	200.9±46.7	199.9±46.3	0.322
	95%-CI	163.7-235.6	164.5-234.7	165.0-236.9	164.4-235.5	
${\dot{\text{V}}}_{\text{E}}$ (L·min^−1^)	m±SD	80.0±14.5	81.6±17.1	82.8±16.9	82.2±15.3	0.326
	95%-CI	68.9-91.2	68.4-94.8	69.8-95.8	70.4-93.9	
V_T_ (L)	m±SD	2.5±0.6	2.5±0.5	2.5±0.6	2.4±0.5	0.328
	95%-CI	2.1-3.0	2.0-2.9	2.1-2.9	2.0-2.8	
f_R_ (min^−1^)	m±SD	32.2±7.7	33.3±6.7	34.0±8.7	34.3±5.9	0.351
	95%-CI	26.3-38.0	28.2-38.4	27.3-40.7	29.8-38.8	
$\dot{\text{V}}{\text{O}}_{2}$ (L·min^−1^)	m±SD	2.6±0.6	2.6±0.6	2.6±0.6	2.6±0.5	0.360
	95%-CI	2.2-3.1	2.1-3.0	2.1-3.1	2.2-3.0	
$\dot{\text{V}}\text{C}{\text{O}}_{2}$ (L·min^−1^)	m±SD	2.8±0.6	2.7±0.6	2.8±0.6	2.8±0.6	0.146
	95%-CI	2.4-3.3	2.3-3.2	2.4-3.3	2.3-3.2	
Heart rate (min^−1^)	m±SD	161.2±5.3	158.0±7.5	157.5±7.1	156.8±7.8	0.009
	95%-CI	157.2-165.2	152.2-163.8	152.1-163.0	150.8-162.8	
Lactate (mmol·L^−1^)	m±SD	6.48±1.22	6.01±1.32	6.14±0.74	6.13±1.15	0.335
	95%-CI	5.54-7.42	4.99-7.02	5.57-6.71	5.25-7.02	
Breathlessness (points)	m±SD	1.1±0.9	0.9±0.6	0.9±0.8	0.9±0.6	0.652
	95%-CI	0.5-1.8	0.4-1.4	0.3-1.5	0.5-1.4	
Respiratory exertion (points)	m±SD	2.7±1.0	1.9±1.1	1.8±1.0	2.2±0.7	0.017
	95%-CI	1.9-3.4	1.0-2.8	1.1-2.6	1.7-2.8	
Leg exertion (points)	m±SD	3.3±1.1	3.3±1.4	3.2±1.1	3.5±1.0	0.877
	95%-CI	2.4-4.1	2.2-4.4	2.4-4.1	2.7-4.3	

Data (n = 9) are presented as mean ± standard deviation (m±SD) and 95% confidence intervals (95%-CI). No significant post-hoc differences were present for any variables between conditions. CON: control warm-up; WU50: hyperpnea at 50% maximal voluntary ventilation (MVV); WU80/30: hyperpnea at 80 and 30% MVV; WU70: hyperpnea at 70% MVV; ${\dot{\text{V}}}_{\text{E}}$, minute ventilation; V_T_, tidal volume; f_R_, respiratory frequency; $\dot{\text{V}}{\text{O}}_{2}$, oxygen consumption; $\dot{\text{V}}\text{C}{\text{O}}_{2}$, carbon dioxide production.

### Changes after the ECh

The maximal decline in FEV_1_ after ECh did not differ between CON (-13.8%, 95% CI: -16.1–-11.4%) and SHAM (-11.3%, 95% CI: -16.9–-5.6%, p = 0.215). However, three out of nine subjects showed a substantial attenuation in the maximal decline in FEV_1_ after ECh in the SHAM condition compared to CON (difference in ΔFEV_1_ between SHAM and CON: 9.0±0.8% [n = 3] versus -0.7±3.4% [n = 6]; S1 Fig). This difference was similar to differences observed after WU50 (6.5±4.0% [n = 3]), WU80/30 (9.3±1.9% [n = 3]) and WU70 (10.2±2.8% [n = 3]). Possibly, for these three subjects, SHAM maneuvers with tidal volumes similar to WU were sufficient to already show warm-up effects at 10% MVV. To avoid misinterpretation in subsequent analyses, data for SHAM was omitted and WU-conditions were compared to CON. An alternative analysis taking into account the observed heterogeneity given in the SHAM trial is presented in S3 and S4 Figs.

**Fig 2:**
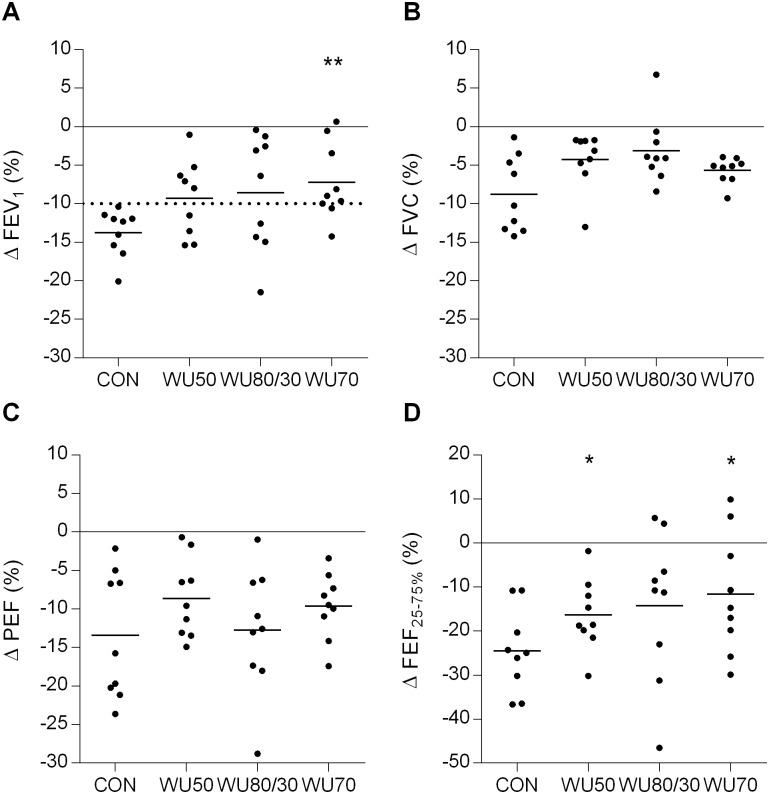
Individual (dots) and mean (line) maximal changes (Δ) from baseline after the exercise challenge in A) forced expiratory volume in 1s (FEV_1_), B) forced vital capacity (FVC), C) peak expiratory flow (PEF) and D) forced expiratory flow between 25 and 75% FVC (FEF_25-75%_) after the different types of 10-min pre-exercise interventions. CON, control warm-up; WU50, hyperpnea at 50% maximal voluntary ventilation (MVV); WU80/30, hyperpnea at 80 and 30% MVV; WU70, hyperpnea at 70% MVV. Dotted line at −10% (FEV_1_) represents a clinically relevant change from baseline. *, ** significantly different from CON (p≤0.05 and p<0.01, respectively).

Maximal changes in FEV_1_, FVC, PEF and FEF_25-75%_ after ECh are given in Fig 2. A main effect of condition was detected for FEV_1_ (p = 0.007), FVC (p = 0.015) and FEF_25-75%_ (p = 0.025), with significantly attenuated maximal decreases in FEV_1_ after WU70 and FEF_25-75%_ after WU50 and WU70 compared to CON. The average decrease in FEV_1_ over the entire recovery period was -8.4% (95% CI: -11.1–-5.7%) following CON, -4.8% (95% CI: -7.9–-1.8%) following WU50, -4.7% (95% CI: -10.0–0.6%) following WU80/30 and -3.6% (95% CI: -7.4–0.3%) following WU70. There was a main effect of condition (p = 0.035) with no significant post-hoc differences.

**Fig 3:**
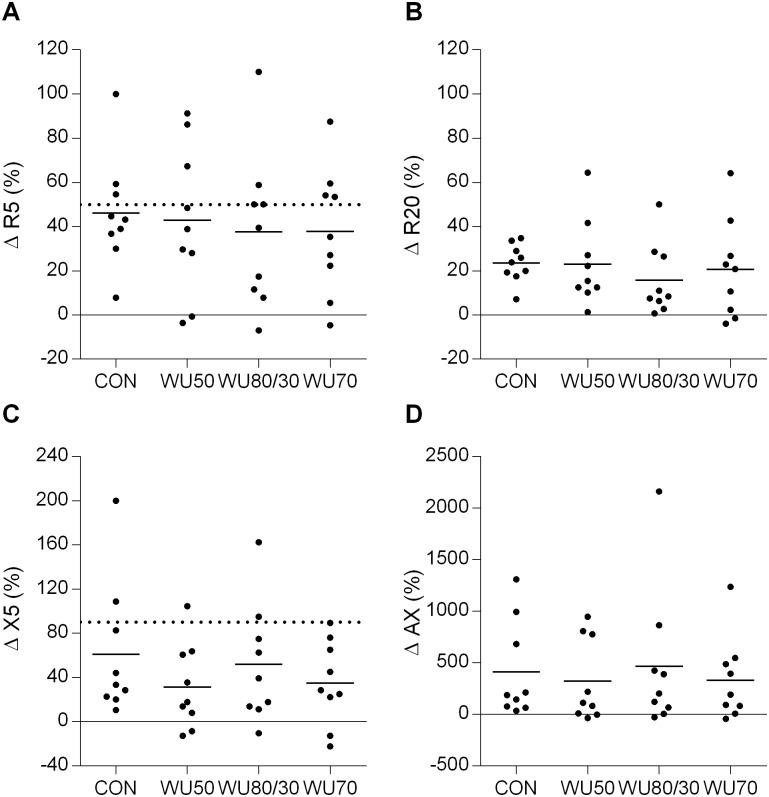
Individual (dots) and mean (line) maximal changes (Δ) from baseline after the exercise challenge in A) airway resistance measured at an impulse frequency of 5Hz (R5), B) airway resistance measured at 20Hz (R20), C) airway reactance measured at 5Hz (X5) and D) reactance area from 5Hz to resonance frequency (AX) after the different types of 10-min pre-exercise interventions. CON, no warm-up; WU50, hyperpnea at 50% maximal voluntary ventilation (MVV); WU80/30, hyperpnea at 80 and 30% MVV; WU70, hyperpnea at 70% MVV. Dotted lines at +50% (R5) and +90% (X5) represent clinically relevant changes from baseline.

Maximal changes in R5, R20, X5 and AX after ECh are depicted in Fig 3. There was no difference between conditions in any of the parameters. Maximal changes in FEV_1_ were only weakly correlated with variables of airway impedance (R5, r = -0.60, p<0.001; R20, rho = -0.45, p = 0.006; X5, rho = -0.47, p = 0.004; AX, rho = -0.52, p = 0.001).

The maximal increase in respiratory exertion after ECh was significantly smaller in WU50 (1.6 points, 95% CI: 0.6–2.5, p = 0.009), WU80/30 (1.6 points, 95% CI: 0.8–2.4, p = 0.009) and WU70 (2.1 points, 95% CI: 1.3–3.0, p = 0.048) compared to CON (3.7 points, 95% CI: 1.9–5.5). The maximal increase in perception of breathlessness did not differ after ECh in WU50 (1.4 points, 95% CI: 0.4–2.4, p = 0.237), WU80/30 (1.9 points, 95% CI: 0.5–3.3, p = 0.258) and WU70 (1.5 points, 95% CI: 0.2–2.8, p = 0.405) compared to CON (2.7 points, 95% CI: 0.7–4.6).

The average degree of bronchoprotection was 32% (WU50, 95% CI: 2–62%), 43% (WU80/30, 95% CI: 7–79%) and 49% (WU70, 95% CI: 22–76%), and did not differ between warm-up conditions (p = 0.451).

In the warm-up trial with the highest degree of bronchoprotection (69%, 95% CI: 50–87%), the 10-min warm-up significantly increased R5 acutely after warm-up (20%, 95% CI: 10–30%) compared to CON (4%, 95% CI: 0–8%, p = 0.020). Furthermore, maximal increases in R5 (p = 0.041), breathlessness (p = 0.043) and respiratory exertion (p = 0.005) were significantly attenuated after the ECh compared to CON. These changes did not result from altered ventilation during the preceding ECh (p = 0.382).

### Effects on time to exhaustion and physiological variables during CET

**Fig 4:**
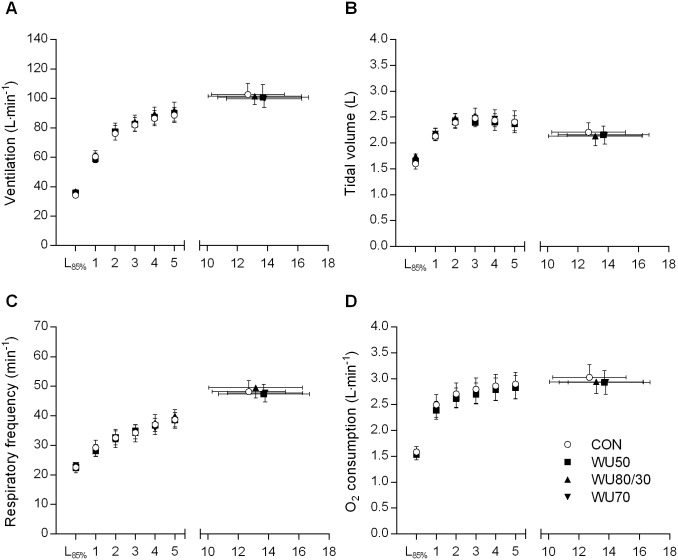
Ventilation, tidal volume, respiratory frequency and oxygen (O_2_) consumption during the endurance test in each condition. Values are mean ± SE. L_85%_, 1min of incremental submaximal stages consisting of 30s at 80% and 30s at 90% of the target workload; CON, no warm-up; WU50, hyperpnea at 50% maximal voluntary ventilation (MVV); WU80/30, hyperpnea at 80 and 30% MVV; WU70, hyperpnea at 70% MVV.

**Fig 5:**
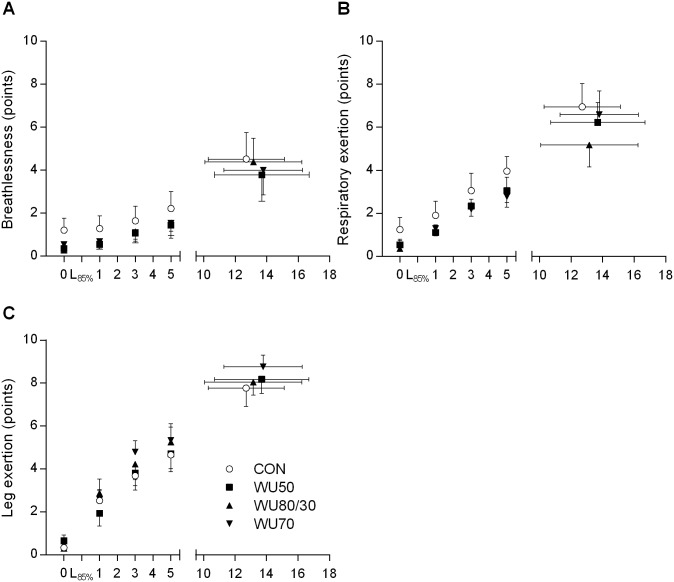
Breathlessness, respiratory exertion and leg exertion during the endurance test in each condition. Values are mean ± SE. 0, before start of exercise; L_85%_, 1min of incremental submaximal stages consisting of 30s at 80% and 30s at 90% of the target workload; CON, no warm-up; WU50, hyperpnea at 50% maximal voluntary ventilation (MVV); WU80/30, hyperpnea at 80 and 30% MVV; WU70, hyperpnea at 70% MVV.

Immediately before CET, FEV_1_ was still significantly reduced in CON (−8.0%, 95% CI: -11.3–-4.7%, p = 0.004) while it did not anymore differ from baseline in the WU50 (-3.8%, 95% CI: -7.9–0.3%, p = 0.300), WU80/30 (-3.1%, 95%CI:-8.7–2.4%, p>1.000) and WU70 (-4.9%, -8.6–-1.2% p = 0.120) condition. Times to exhaustion (p = 0.582), ventilation, gas exchange, subjective measures (Figs 4 and 5), heart rate and blood lactate concentration (both p>0.562) did not differ between conditions. Also, when comparing values of the WU-trial with the individually best FEV_1_ immediately before the start of the CET (3.8 L, 95% CI: 3.2–4.5 L) with CON (3.6 L, 95% CI: 3.1–4.0 L; p = 0.025), ventilation, gas exchange, heart rate, subjective ratings and time to exhaustion were not different from CON (all p>0.069).

## Discussion

Results of the present study suggest that, in mild asthmatics with EIB, pre-exercise hyperpnea of different intensity, using warm and humid air, does not induce bronchoconstriction, may attenuate the decrease in lung function and does not negatively affect performance.

### Effects of pre-exercise hyperpnea on airway function after ECh

Warm-up with physical exercise, inducing EIB by itself, was shown to improve EIB in a subsequent exercise with bronchoprotection ranging from ~6 to 70% [6–11], a level achieved in the present study with isolated hyperpnea as well. The present study thus provides evidence that even non-EIB-inducing and respiratory-only warm-up may induce bronchoprotection.

Positive effects of warm-up on FEV_1_, FVC and FEF_25-75%_were larger and more consistent than those on PEF while previous studies also reported attenuated declines in PEF after exercise warm-up interventions [6,9]. Since PEF is known to be primarily effort-dependent [28], also effort-independent parameters of airway impedance were measured in the present study. However, improvements in impedance with warm-up were minimal and maximal changes correlated only weakly with FEV_1_. Although we need to consider that—due to time restrictions—breaks between spirometry and impulse oscillometry were shorter than recommended [23] and only one single oscillometry measurement was performed per time-point in the breaks. This might have resulted in higher airway impedance or less attenuation compared to lege-artis measurements since deep inspirations (as performed during spirometry) are known to increase airway resistance in asthmatics per se [29]. However, when selecting the best trial in terms of the bronchoprotection index, the maximal increase in airway resistance after ECh was significantly smaller than in CON. Also, those subjects with pathological increases in R5 and X5 following CON showed improvements in the warm-up trials, reaching levels below clinical thresholds in most cases. Thus with more severe airway dysfunction, improvements in impedance can be expected to be more pronounced and consistent.

Asthma symptoms, e.g. cough, wheeze, chest tightness, shortness of breath, or excessive mucous production occur frequently in asthmatics in the context of exercise [30,31]. Whether warm-up exercises aiming at attenuating EIB has also effects on asthma symptoms is unclear but might be expected. We are aware of only one study reporting significantly less wheezing after exercise with warm-up compared to the exercise without [7]. Data from the present study suggest only minimal effects of warm-up on perception of breathlessness and respiratory exertion *during* the ECh while perception of respiratory exertion was significantly reduced *after* the ECh in all WU-trials. Additionally, in the subgroup analysis considering only the trial with the greatest degree of bronchoprotection, both a significantly reduced perception of breathlessness and respiratory exertion were observed after ECh. Given the high prevalence of exercise-induced respiratory symptoms and their potential association with avoidance of physical activity [30], factors reducing these symptoms certainly deserve further investigations.

In the present study, moderate-, alternating (high/low)- and high-intensity warm-up strategies were chosen based on previous findings with hyperpnea [14,32] and with physical exercise warm-up trials [8,10]. According to a recent meta-analysis, most pronounced effects were expected to occur after interval and variable intensity respiratory warm-up since both continuous low- and high-intensity warm-up did not provide significant improvements [13]. However, this meta-analysis was based on studies using different protocols in different groups of subjects. Only two studies varied warm-up intensity without changing the duration of the warm-up or the break prior to the next exercise [8,11] and both found no significant effects after interval exercise but attenuated EIB after continuous moderate or low-intensity running—a finding which was not seen in the meta-analysis. In the present study, however, where timing was kept similar, only the continuous respiratory exercise at high intensity offered significant bronchoprotection while interval as well as continuous respiratory exercise at moderate intensity showed improvements only by tendency. Thus, the modality and protocol with the best bronchoprotective effect still remain to be established.

Regarding potentially different effects of different warm-up modalities, bronchoprotective prostaglandins (e.g. PGE2) need to be considered. In fact, pre-treatment with COX-inhibitors (e.g. indomethacine) significantly abolished refractoriness to repeated physical exercise, independent of intensity or the condition of inspired air [17,33]. No such inhibitor-induced suppression was observed in a study with repeated hyperpnea challenges [34], suggesting that exercise per se and not the level of ventilation might trigger release of protective PGs. This is, however, challenged by an in-vitro study where cyclic stretch of airway epithelial cells increased PGE2 release in a frequency-dependent manner [35] and also by a recent study that showed increased PGE2 concentrations after hyperpnea and significant refractoriness after a subsequent second hyperpnea [36]. It thus seems conceivable that, in the present study, hyperpnea but not NWU stretched the airways enough to release PGE2, reduce EIB severity and improve lung function recovery, possibly via blocking leukotriene-receptors on airway endothelial cells, thereby inhibiting their bronchoconstrictive effects [37]. Reduced EIB severity and improved lung function recovery were previously shown after selectively antagonizing histamine and leukotriene [38]. The fact that airway resistance was increased after hyperpnea in warm-up trials with the highest degree of bronchoprotection, also points towards this mechanism. This finding confirms previous results where an interplay of bronchoconstrictive and bronchoprotective mediators was suggested to promote refractoriness [39].

### Effects of pre-exercise hyperpnea on exercise performance

In the present study, we also aimed to assess whether intense pre-exercise hyperpnea would negatively affect exercise performance compared to exercise without prior hyperpnea. With the present design we did not observe a negative effect of the respiratory work performed during warm-up on time to exhaustion. However, we need to consider one caveat: Ideally the CET would have needed to follow hyperpnea directly (without the ECh in between), which would have meant that subjects needed to perform another 5 tests on independent days. We therefore opted for the present compromise. This might have influenced the outcome in several ways: i) exercise-induced respiratory muscle fatigue could have developed in the ECh since ventilation in the ECh (~58% MVV) was similar to the level that induced respiratory muscle fatigue in a previous study (~54% MVV; [40]) which would have compromised performance; ii) potential hyperpnea-induced respiratory muscle fatigue might have recovered during the ECh (as previously observed–[41]) or in the 30-min break between cycling tests which would have masked a fatigue-induced impairment in CET performance; and iii) ECh itself could have induced a refractory period resulting in decreased airway obstruction such that performance after CON was better than it would have been without the ECh. However, also larger degrees of bronchoconstriction prior to a CET would likely not affect performance as previously shown in a similar setting (-27±15%; [42]).

## Conclusion

The present data suggest that intense pre-exercise hyperpnea with partial rebreathing that does not, by itself, induce EIB, significantly attenuates bronchoconstriction and improves lung function recovery after an exercise challenge while performance is not affected by the intense pre-exercise hyperpnea.

## Supporting Information

S1 Fig Maximal changes in lung function after the exercise challenge in the different experimental conditions, including control and sham. (PDF)

S2 Fig Maximal changes in airway impedance after the exercise challenge in the different experimental conditions, including control and sham. (PDF)

S3 Fig Alternative analysis of the maximal changes in lung function after the exercise challenge in the different experimental conditions. (PDF)

S4 Fig Alternative analysis of the maximal changes in airway impedance after the exercise challenge in the different experimental conditions. (PDF)

S1 Table Baseline lung function and airway impedance in the different experimental conditions. (PDF)

S2 Table Maximal changes in lung function and airway impedance immediately after warm-up in the different experimental conditions. (PDF)

## References

[1] Franca-Pinto A, Mendes FA, de Carvalho-Pinto RM, Agondi RC, Cukier A, Stelmach R, et al Aerobic training decreases bronchial hyperresponsiveness and systemic inflammation in patients with moderate or severe asthma: a randomised controlled trial. Thorax. 2015;70: 732–739. 10.1136/thoraxjnl-2014-206070 26063507

[2] Eichenberger PA, Diener SN, Kofmehl R, Spengler CM. Effects of exercise training on airway hyperreactivity in asthma: a systematic review and meta-analysis. Sports Med. 2013;43: 1157–1170. 10.1007/s40279-013-0077-2 23846823

[3] Weiler JM, Anderson SD, Randolph C, Bonini S, Craig TJ, Pearlman DS, et al Pathogenesis, prevalence, diagnosis, and management of exercise-induced bronchoconstriction: a practice parameter. Ann Allergy Asthma Immunol. 2010;105: S1–47. 10.1016/j.anai.2010.09.021 21167465

[4] Edmunds AT, Tooley M, Godfrey S. The refractory period after exercise-induced asthma: its duration and relation to the severity of exercise. Am Rev Respir Dis. 1978;117: 247–254. 10.1164/arrd.1978.117.2.247 637407

[5] McNeill RS, Nairn JR, Millar JS, Ingram CG. Exercise-induced asthma. Q J Med. 1966;35: 55–67. 4380323

[6] Schnall RP, Landau LI. Protective effects of repeated short sprints in exercise-induced asthma. Thorax. 1980;35: 828–832. 7221978 10.1136/thx.35.11.828 PMC471392

[7] Reiff DB, Choudry NB, Pride NB, Ind PW. The effect of prolonged submaximal warm-up exercise on exercise-induced asthma. Am Rev Respir Dis. 1989;139: 479–484. 10.1164/ajrccm/139.2.479 2913893

[8] McKenzie DC, McLuckie SL, Stirling DR. The protective effects of continuous and interval exercise in athletes with exercise-induced asthma. Med Sci Sports Exerc. 1994;26: 951–956. 7968428

[9] de Bisschop C, Guenard H, Desnot P, Vergeret J. Reduction of exercise-induced asthma in children by short, repeated warm ups. Br J Sports Med. 1999;33: 100–104. 10205690 10.1136/bjsm.33.2.100 PMC1756157

[10] Mickleborough TD, Lindley MR, Turner LA. Comparative effects of a high-intensity interval warm-up and salbutamol on the bronchoconstrictor response to exercise in asthmatic athletes. Int J Sports Med. 2007;28: 456–462. 10.1055/s-2006-924583 17111314

[11] Eck R, Lachtermann E, Pleyer K, Schmitz M, Jung K. Effect of standardized warm-up programs on the intensity and frequency of exercise induced asthma (EIA) in children and teenagers [in German]. Mainzer sportmedizinische Schriftenreihe. 2001;3: 97–108.

[12] Morton AR, Fitch KD, Davis T. The effect of "warm-up" on exercise-induced asthma. Ann Allergy. 1979;42: 257–260. 434587

[13] Stickland MK, Rowe BH, Spooner CH, Vandermeer B, Dryden DM. Effect of warm-up exercise on exercise-induced bronchoconstriction. Med Sci Sports Exerc. 2012;44: 383–391. 10.1249/MSS.0b013e31822fb73a 21811185

[14] Ben-Dov I, Gur I, Bar-Yishay E, Godfrey S. Refractory period following induced asthma: contributions of exercise and isocapnic hyperventilation. Thorax. 1983;38: 849–853. 6648867 10.1136/thx.38.11.849 PMC459675

[15] Hancox RJ, Subbarao P, Kamada D, Watson RM, Hargreave FE, Inman MD. Beta2-agonist tolerance and exercise-induced bronchospasm. Am J Respir Crit Care Med. 2002;165: 1068–1070. 10.1164/ajrccm.165.8.200111-091bc 11956046

[16] Inman MD, O'Byrne PM. The effect of regular inhaled albuterol on exercise-induced bronchoconstriction. Am J Respir Crit Care Med. 1996;153: 65–69. 10.1164/ajrccm.153.1.8542164 8542164

[17] Wilson BA, Bar-Or O, O'Byrne PM. The effects of indomethacin on refractoriness following exercise both with and without a bronchoconstrictor response. Eur Respir J. 1994;7: 2174–2178. 7713200 10.1183/09031936.94.07122174

[18] Bolger C, Tufvesson E, Anderson SD, Devereux G, Ayres JG, Bjermer L, et al Effect of inspired air conditions on exercise-induced bronchoconstriction and urinary CC16 levels in athletes. J Appl Physiol (1985). 2011;111: 1059–1065. 21799131 10.1152/japplphysiol.00113.2011

[19] Renggli AS, Verges S, Notter DA, Spengler CM. Development of respiratory muscle contractile fatigue in the course of hyperpnoea. Respir Physiol Neurobiol. 2008;164: 366–372. 10.1016/j.resp.2008.08.008 18801466

[20] Romer LM, Polkey MI. Exercise-induced respiratory muscle fatigue: implications for performance. J Appl Physiol. 2008;104: 879–888. 10.1152/japplphysiol.01157.2007 18096752

[21] GINA. The global strategy for asthma management and prevention, Global Initiative for Asthma (GINA). http://www.ginasthma.org/ [accessed 2016 June 16]. 2016.

[22] Crapo RO, Casaburi R, Coates AL, Enright PL, Hankinson JL, Irvin CG, et al Guidelines for methacholine and exercise challenge testing-1999. This official statement of the American Thoracic Society was adopted by the ATS Board of Directors, July 1999. Am J Respir Crit Care Med. 2000;161: 309–329. 10.1164/ajrccm.161.1.ats11-99 10619836

[23] Oostveen E, MacLeod D, Lorino H, Farre R, Hantos Z, Desager K, et al The forced oscillation technique in clinical practice: methodology, recommendations and future developments. Eur Respir J. 2003;22: 1026–1041. 14680096 10.1183/09031936.03.00089403

[24] Miller MR, Hankinson J, Brusasco V, Burgos F, Casaburi R, Coates A, et al Standardisation of spirometry. Eur Respir J. 2005;26: 319–338. 10.1183/09031936.05.00034805 16055882

[25] Quanjer PH, Tammeling GJ, Cotes JE, Pedersen OF, Peslin R, Yernault JC. Lung volumes and forced ventilatory flows. Report Working Party Standardization of Lung Function Tests, European Community for Steel and Coal. Official Statement of the European Respiratory Society. Eur Respir J. 1993;16: 5–40. 8499054

[26] Quanjer PH, Stanojevic S, Cole TJ, Baur X, Hall GL, Culver BH, et al Multi-ethnic reference values for spirometry for the 3-95-yr age range: the global lung function 2012 equations. Eur Respir J. 2012;40: 1324–1343. 10.1183/09031936.00080312 22743675 PMC3786581

[27] Dahlen B, O'Byrne PM, Watson RM, Roquet A, Larsen F, Inman MD. The reproducibility and sample size requirements of exercise-induced bronchoconstriction measurements. Eur Respir J. 2001;17: 581–588. 11401049 10.1183/09031936.01.17405810

[28] Mead J. Analysis of the configuration of maximum expiratory flow-volume curves. J Appl Physiol Respir Environ Exerc Physiol. 1978;44: 156–165. 632154 10.1152/jappl.1978.44.2.156

[29] How SC, Romer LM, McConnell AK. Acute effects of inspiratory pressure threshold loading upon airway resistance in people with asthma. Respir Physiol Neurobiol. 2009;166: 159–163. 10.1016/j.resp.2009.03.003 19442932

[30] Parsons JP, Craig TJ, Stoloff SW, Hayden ML, Ostrom NK, Eid NS, et al Impact of exercise-related respiratory symptoms in adults with asthma: Exercise-Induced Bronchospasm Landmark National Survey. Allergy Asthma Proc. 2011;32: 431–437. 10.2500/aap.2011.32.3501 22221437

[31] Rundell KW, Im J, Mayers LB, Wilber RL, Szmedra L, Schmitz HR. Self-reported symptoms and exercise-induced asthma in the elite athlete. Med Sci Sports Exerc. 2001;33: 208–213. 11224807 10.1097/00005768-200102000-00006

[32] Tweeddale PM, Godden DJ, Grant IW. Hyperventilation or exercise to induce asthma? Thorax. 1981;36: 596–598. 7314034 10.1136/thx.36.8.596 PMC471641

[33] Wilson BA. Effects of indomethacin on refractoriness when exercise intensity is graded to prevent exercise asthma (1078). Med Sci Sports Exerc. 1996;28: S181.

[34] Margolskee DJ, Bigby BG, Boushey HA. Indomethacin blocks airway tolerance to repetitive exercise but not to eucapnic hyperpnea in asthmatic subjects. Am Rev Respir Dis. 1988;137: 842–846. 10.1164/ajrccm/137.4.842 3354990

[35] Savla U, Sporn PH, Waters CM. Cyclic stretch of airway epithelium inhibits prostanoid synthesis. Am J Physiol. 1997;273: L1013–1019. 9374729 10.1152/ajplung.1997.273.5.L1013

[36] Bood JR, Sundblad BM, Delin I, Sjodin M, Larsson K, Anderson SD, et al Urinary excretion of lipid mediators in response to repeated eucapnic voluntary hyperpnea in asthmatic subjects. J Appl Physiol (1985). 2015;119: 272–279. 26112240 10.1152/japplphysiol.00301.2015

[37] Larsson J, Anderson SD, Dahlen SE, Dahlen B. Refractoriness to exercise challenge: a review of the mechanisms old and new. Immunol Allergy Clin North Am. 2013;33: 329–345, viii 10.1016/j.iac.2013.02.004 23830128

[38] Currie GP, Haggart K, Lee DK, Fowler SJ, Wilson AM, Brannan JD, et al Effects of mediator antagonism on mannitol and adenosine monophosphate challenges. Clin Exp Allergy. 2003;33: 783–788. 12801313 10.1046/j.1365-2222.2003.01688.x

[39] Larsson J, Perry CP, Anderson SD, Brannan JD, Dahlen SE, Dahlen B. The occurrence of refractoriness and mast cell mediator release following mannitol-induced bronchoconstriction. J Appl Physiol (1985). 2011;110: 1029–1035. 21252215 10.1152/japplphysiol.00978.2010

[40] Verges S, Lenherr O, Haner AC, Schulz C, Spengler CM. Increased fatigue resistance of respiratory muscles during exercise after respiratory muscle endurance training. Am J Physiol Regul Integr Comp Physiol. 2007;292: R1246–1253. 10.1152/ajpregu.00409.2006 17068160

[41] Wuthrich TU, Notter DA, Spengler CM. Effect of inspiratory muscle fatigue on exercise performance taking into account the fatigue-induced excess respiratory drive. Exp Physiol. 2013;98: 1705–1717. 10.1113/expphysiol.2013.073635 24014807

[42] Rossman MJ, Nader S, Berry D, Orsini F, Klansky A, Haverkamp HC. Effects of altered airway function on exercise ventilation in asthmatic adults. Med Sci Sports Exerc. 2014;46: 1104–1113. 10.1249/MSS.0000000000000206 24576858 PMC4028423

