# Supplementary material for: Pre-Exercise Hyperpnea Attenuates Exercise-Induced Bronchoconstriction Without Affecting Performance
Source: PLoS One. 2016 Nov 29;11(11):e0167318. doi: 10.1371/journal.pone.0167318 (PMC5127560; doi:10.1371/journal.pone.0167318)
Supplement: S1 Fig — (PDF) [file pone.0167318.s001.pdf]

Maximal changes in lung function after the exercise challenge in the different experimental conditions, including control and sham

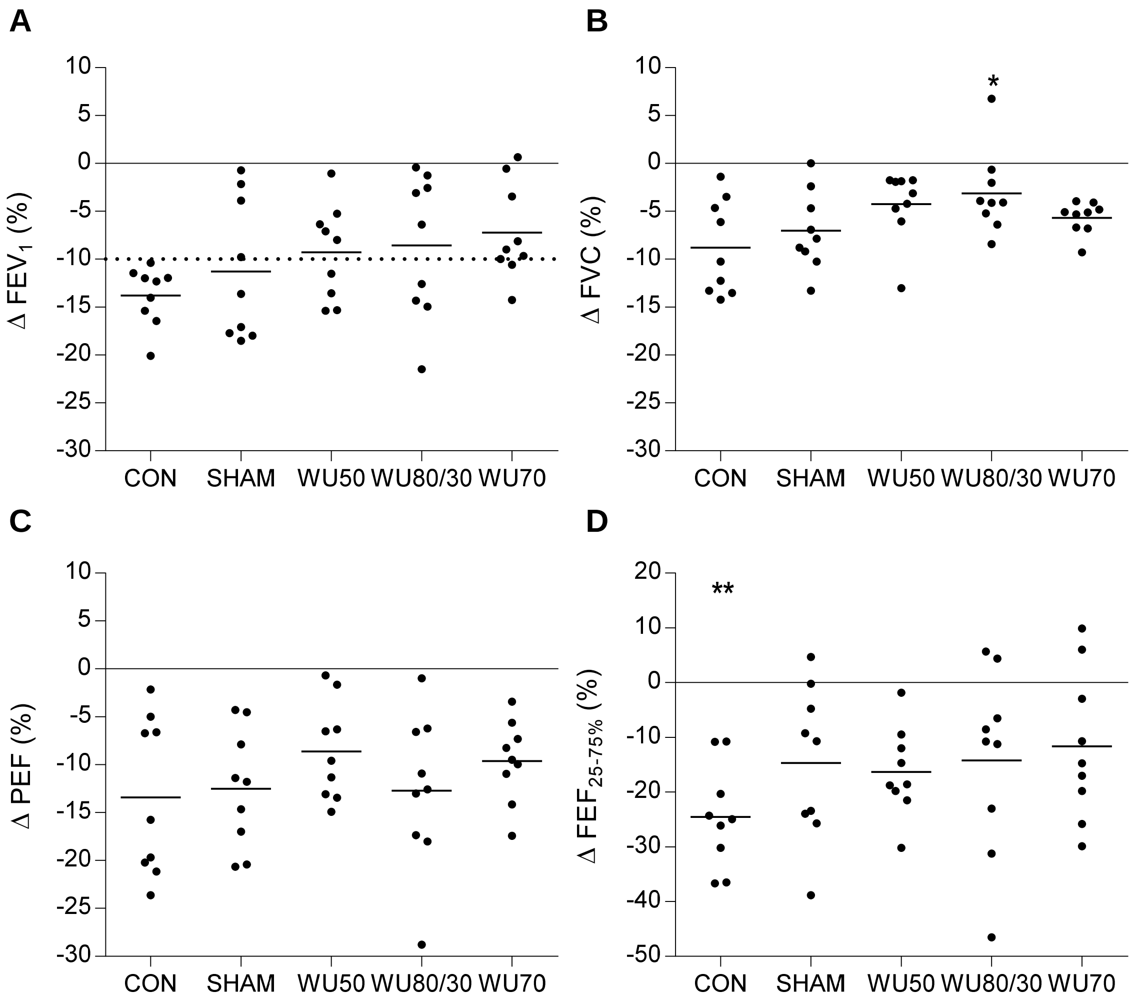

S1 Fig. Individual (dots) and mean (line) maximal changes (Δ) from baseline after the exercise challenge in A) forced expiratory volume in 1s (FEV_1), B) forced vital capacity (FVC), C) peak expiratory flow (PEF) and D) forced expiratory flow between 25 and 75% FVC ($FEF_{25-75\%}$) after the different types of 10-min pre-exercise interventions. CON, control warm-up; SHAM, hyperpnea at 10% maximal voluntary ventilation (MVV), WU50, hyperpnea at 50% MVV; WU80/30, hyperpnea at 80 and 30% MVV; WU70, hyperpnea at 70% MVV. Dotted line at −10% (FEV_1) represents a clinically relevant change from baseline. *, ** significantly different from SHAM ($p \leq 0.05$ and $p < 0.01$, respectively) using one-way ANOVA with repeated measures and Bonferroni post-hoc adjustments.
