# Supplementary material for: Pre-Exercise Hyperpnea Attenuates Exercise-Induced Bronchoconstriction Without Affecting Performance
Source: PLoS One. 2016 Nov 29;11(11):e0167318. doi: 10.1371/journal.pone.0167318 (PMC5127560; doi:10.1371/journal.pone.0167318)
Supplement: S2 Fig — (PDF) [file pone.0167318.s002.pdf]

Maximal changes in airway impedance after the exercise challenge in the different experimental conditions, including control and sham

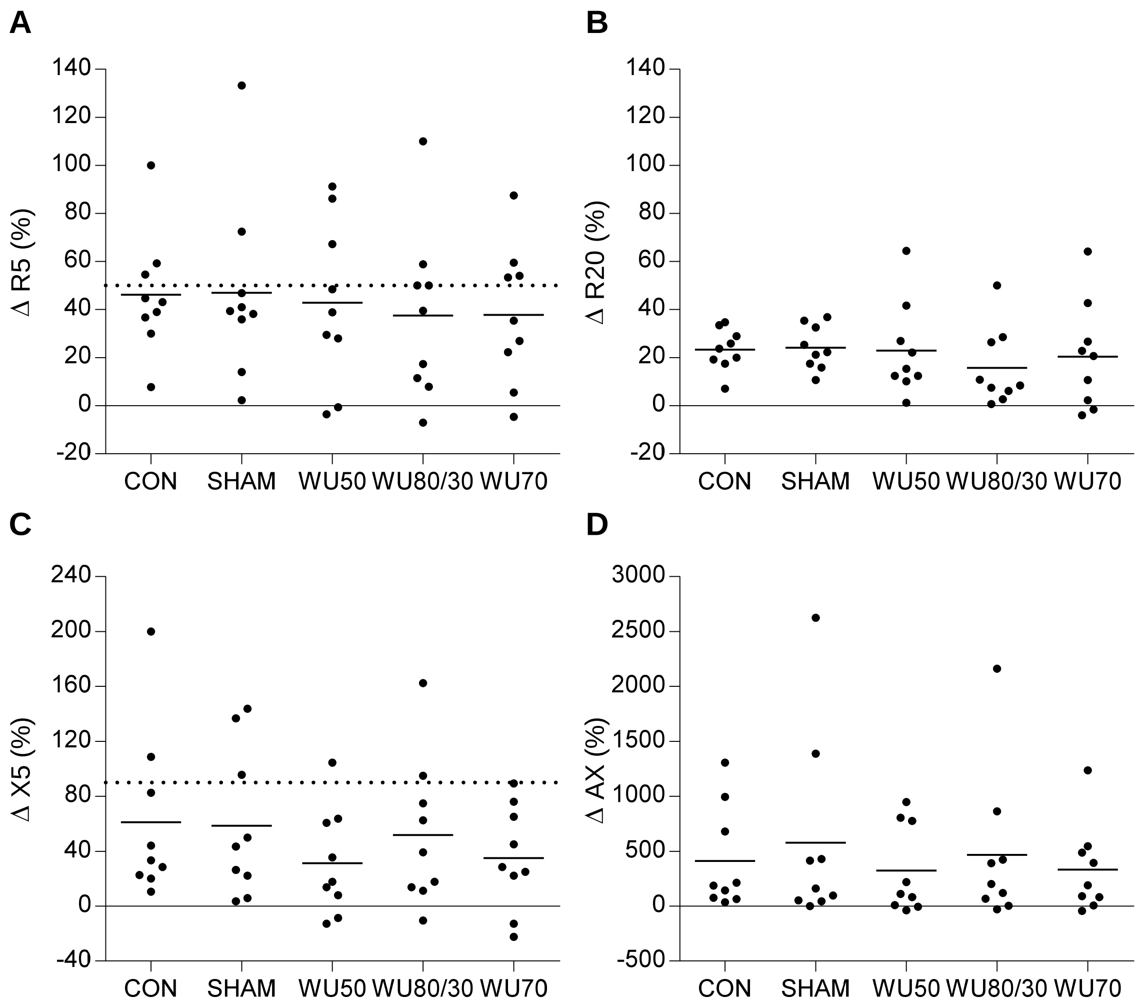

S2 Fig. Individual (dots) and mean (line) maximal changes (Δ) from baseline after the exercise challenge in A) airway resistance measured at an impulse frequency of 5Hz (R5), B) airway resistance measured at 20Hz (R20), C) airway reactance measured at 5Hz (X5) and D) reactance area from 5Hz to resonance frequency (AX) after the different types of 10-min pre-exercise interventions. CON, no warm-up; SHAM, hyperpnea at 10% maximal voluntary ventilation (MVV), WU50, hyperpnea at 50% MVV; WU80/30, hyperpnea at 80 and 30% MVV; WU70, hyperpnea at 70% MVV. Dotted lines at +50% (R5) and +90% (X5) represent clinically relevant changes from baseline.
