# Supplementary material for: Pre-Exercise Hyperpnea Attenuates Exercise-Induced Bronchoconstriction Without Affecting Performance
Source: PLoS One. 2016 Nov 29;11(11):e0167318. doi: 10.1371/journal.pone.0167318 (PMC5127560; doi:10.1371/journal.pone.0167318)
Supplement: S4 Fig — (PDF) [file pone.0167318.s004.pdf]

Alternative analysis of the maximal changes in airway impedance after the exercise challenge in the different experimental conditions

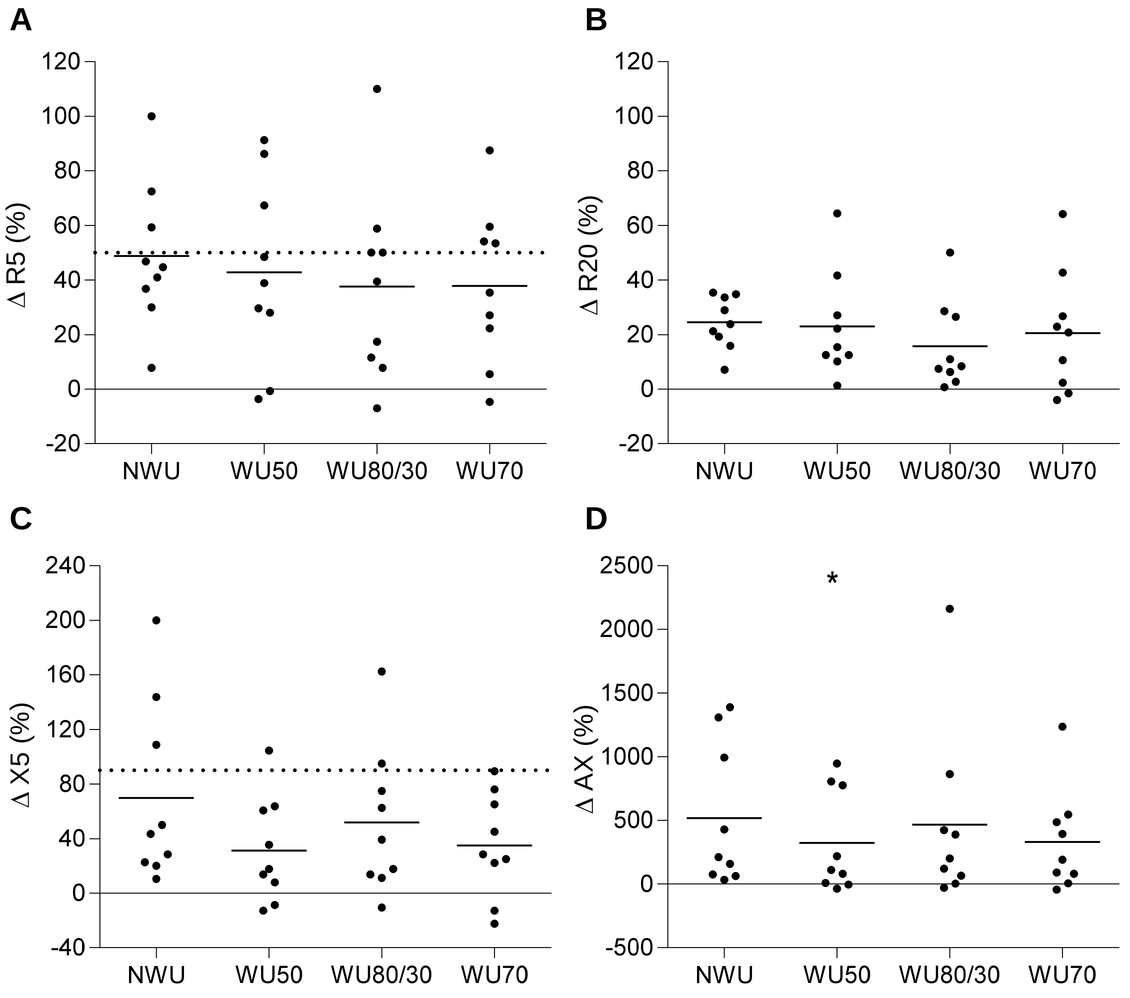

S4 Fig. Individual (dots) and mean (line) maximal changes (Δ) from baseline after the exercise challenge in A) airway resistance measured at an impulse frequency of 5Hz (R5), B) airway resistance measured at 20Hz (R20), C) airway reactance measured at 5Hz (X5) and D) reactance area from 5Hz to resonance frequency (AX) after the different types of 10-min pre-exercise interventions. NWU, no warm-up, i.e. control warm-up (CON) or hyperpnea at 10% maximal voluntary ventilation (MVV, SHAM), consisting of the trial with the more pronounced decrease in FEV_1 after exercise challenge; WU50, hyperpnea at 50% MVV; WU80/30, hyperpnea at 80 and 30% MVV; WU70, hyperpnea at 70% MVV. Dotted lines at +50% (R5) and +90% (X5) represent clinically relevant changes from baseline. * significantly different from NWU (p≤0.05) using one-way ANOVA with repeated measures and Bonferroni post-hoc adjustments.
