# Supplementary material for: Pre-Exercise Hyperpnea Attenuates Exercise-Induced Bronchoconstriction Without Affecting Performance
Source: PLoS One. 2016 Nov 29;11(11):e0167318. doi: 10.1371/journal.pone.0167318 (PMC5127560; doi:10.1371/journal.pone.0167318)
Supplement: S1 Table — (PDF) [file pone.0167318.s005.pdf]

S1 Table. Baseline lung function and airway impedance in the different experimental conditions

		CON	SHAM	WU50	WU80/30	WU70	p-value
FVC (L)	m±SD	5.06 ± 0.84	5.02 ± 0.76	5.04 ± 0.83	5.06 ± 0.87	5.05 ± 0.90	0.931
	95%-CI	4.41 - 5.71	4.43 - 5.60	4.40 - 5.68	4.39 - 5.73	4.35 - 5.74	
FVC (%pred)	m±SD	113 ± 21	112 ± 20	112 ± 20	113 ± 21	112 ± 20	0.944
	95%-CI	97 - 129	96 - 127	97 - 127	96 - 129	97 - 128	
FEV_1 (L)	m±SD	3.90 ± 0.73	3.83 ± 0.72	3.87 ± 0.73	3.86 ± 0.74	3.83 ± 0.76	0.578
	95%-CI	3.34 - 4.46	3.28 - 4.39	3.31 - 4.43	3.29 - 4.42	3.25 - 4.42	
FEV_1 (%pred)	m±SD	101 ± 16	100 ± 16	101 ± 15	100 ± 16	99 ± 16	0.529
	95%-CI	89 - 114	87 - 112	89 - 112	88 - 113	87 - 112	
FEV_1/FVC (%)	m±SD	77.0 ± 6.6	76.3 ± 7.0	77.2 ± 7.4	76.1 ± 7.3	76.2 ± 6.6	0.577
	95%-CI	71.9 - 82.1	70.9 - 81.7	71.5 - 82.9	70.5 - 81.7	71.1 - 81.2	
FEV_1/FVC (%pred)	m±SD	90 ± 8	89 ± 9	90 ± 9	89 ± 9	89 ± 8	0.584
	95%-CI	84 - 96	82 - 96	83 - 97	82 - 96	83 - 95	
PEF ($L \cdot s^{-1}$)	m±SD	8.33 ± 1.86	8.44 ± 1.53	8.33 ± 1.59	8.49 ± 1.63	8.42 ± 1.39	0.787
	95%-CI	6.90 - 9.76	7.27 - 9.62	7.11 - 9.56	7.23 - 9.74	7.35 - 9.48	
PEF (%pred)	m±SD	101 ± 16	104 ± 19	102 ± 17	104 ± 17	103 ± 14	0.575
	95%-CI	89 - 113	89 - 118	89 - 115	91 - 117	92 - 114	
$FEF_{25\text{-}75\%}$ ($L \cdot s^{-1}$)	m±SD	3.32 ± 1.13	3.14 ± 1.05	3.22 ± 1.01	3.20 ± 1.12	3.15 ± 0.99	0.534
	95%-CI	2.45 - 4.19	2.33 - 3.95	2.44 - 3.99	2.34 - 4.06	2.38 - 3.91	
$FEF_{25\text{-}75\%}$ (%pred)	m±SD	73 ± 20	69 ± 19	71 ± 18	71 ± 20	69 ± 18	0.528
	95%-CI	58 - 88	54 - 84	57 - 85	55 - 86	56 - 83	
R5 ($kPa \cdot L^{-1} \cdot s^{-1}$)	m±SD	0.33 ± 0.10	0.31 ± 0.08	0.33 ± 0.09	0.33 ± 0.07	0.33 ± 0.07	0.620
	95%-CI	0.25 - 0.40	0.25 - 0.37	0.26 - 0.40	0.28 - 0.38	0.28 - 0.38	
R20 ($kPa \cdot L^{-1} \cdot s^{-1}$)	m±SD	0.32 ± 0.08	0.31 ± 0.06	0.32 ± 0.06	0.32 ± 0.05	0.32 ± 0.05	0.539
	95%-CI	0.26 - 0.38	0.26 - 0.35	0.27 - 0.37	0.28 - 0.36	0.28 - 0.36	
R5-R20 ($kPa \cdot L^{-1} \cdot s^{-1}$)	m±SD	0.01 ± 0.03	0.01 ± 0.03	0.01 ± 0.04	0.01 ± 0.04	0.01 ± 0.03	0.531
	95%-CI	-0.02 - 0.03	-0.02 - 0.03	-0.02 - 0.04	-0.02 - 0.04	-0.01 - 0.04	
X5 ($kPa \cdot L^{-1} \cdot s^{-1}$)	m±SD	-0.10 ± 0.04	-0.09 ± 0.03	-0.10 ± 0.04	-0.09 ± 0.03	-0.10 ± 0.04	0.010
	95%-CI	-0.13 - -0.07	-0.11 - -0.06	-0.13 - -0.07	-0.11 - -0.06	-0.13 - -0.06	
AX ($kPa \cdot L^{-1}$)	m±SD	0.35 ± 0.55	0.28 ± 0.40	0.34 ± 0.50	0.30 ± 0.42	0.32 ± 0.43	0.266
	95%-CI	-0.08 - 0.77	-0.03 - 0.59	-0.04 - 0.72	-0.02 - 0.62	-0.01 - 0.65	

Data (n=9) are presented as mean ± standard deviation (m ± SD) and 95% confidence intervals (95%-CI). CON: control warm-up; SHAM: hyperpnea at 10% maximal voluntary ventilation (MVV); WU50: hyperpnea at 50% MVV; WU80/30: hyperpnea at 80 and 30% MVV; WU70: hyperpnea at 70% MVV; FVC: forced vital capacity; FEV_1: forced expiratory volume in 1s; PEF: peak expiratory flow; $FEF_{25\text{-}75\%}$: forced expiratory flow between 25 and 75% FVC; R5: airway resistance at 5Hz; R20: airway resistance at 20Hz; R5-R20: difference in airway resistance measured at 5 and 20Hz; X5: airway reactance at 5Hz; AX: airway reactance area from 5Hz to resonance frequency; pred: predicted. All post-hoc comparisons were not significantly different from CON using one-way ANOVA with repeated measures and Bonferroni post-hoc adjustments.
