# Supplementary material for: Pre-Exercise Hyperpnea Attenuates Exercise-Induced Bronchoconstriction Without Affecting Performance
Source: PLoS One. 2016 Nov 29;11(11):e0167318. doi: 10.1371/journal.pone.0167318 (PMC5127560; doi:10.1371/journal.pone.0167318)
Supplement: S2 Table — (PDF) [file pone.0167318.s006.pdf]

S2 Table. Maximal changes acutely after warm-up in lung function and airway impedance in the different experimental conditions

		CON	SHAM	WU50	WU80/30	WU70	p-value
Δ FVC (%)	m±SD	-3.6 ± 2.2	-3.6 ± 2.4	-4.4 ± 3.0	-2.4 ± 5.5	-4.4 ± 3.2	0.538
	95%-CI	-5.3 - -1.9	-5.4 - -1.8	-6.7 - -2.1	-6.6 - 1.8	-6.9 - -1.9	
Δ FEV_1 (%)	m±SD	-2.5 ± 1.9	-2.6 ± 2.2	-5.1 ± 2.0	-3.3 ± 4.3	-4.1 ± 3.5	0.214
	95%-CI	-4.0 - -1.0	-4.3 - -0.9	-6.6 - -3.5	-6.6 - 0.0	-6.8 - -1.4	
Δ PEF (%)	m±SD	-4.6 ± 5.5	-4.3 ± 3.0	-4.3 ± 2.7	-3.0 ± 1.4	-5.7 ± 3.3	0.533
	95%-CI	-8.9 - -0.4	-6.7 - -2.0	-6.4 - -2.2	-4.1 - -1.9	-8.2 - -3.2	
Δ $FEF_{25-75\%}$ (%)	m±SD	-2.2 ± 6.8	0.9 ± 5.5	-7.6 ± 7.8	-5.4 ± 5.9	-6.5 ± 7.1	0.048
	95%-CI	-7.5 - 3.0	-3.4 - 5.1	-13.6 - -1.5	-10.0 - -0.9	-11.9 - -1.1	
Δ R5 (%)	m±SD	4.2 ± 5.0	14.7 ± 9.1#	23.2 ± 19.0	19.0 ± 11.9#	17.0 ± 13.1	0.016
	95%-CI	0.4 - 8.1	7.7 - 21.7	8.5 - 37.8	9.8 - 28.1	7.0 - 27.1	
Δ R20 (%)	m±SD	2.6 ± 5.2	11.8 ± 8.2	13.9 ± 17.7	5.3 ± 5.7	8.7 ± 13.2	0.169
	95%-CI	-1.5 - 6.6	5.4 - 18.1	0.3 - 27.5	0.9 - 9.7	-1.5 - 18.9	
Δ X5 (%)	m±SD	15.4 ± 8.7	21.2 ± 17.8	23.9 ± 23.1	33.4 ± 30.9	21.3 ± 13.6	0.150
	95%-CI	8.7 - 22.0	7.5 - 35.0	6.1 - 41.7	9.7 - 57.1	10.8 - 31.8	
Δ AX (%)	m±SD	31.9 ± 19.2	59.5 ± 56.1	108.4 ± 125.5	189.1 ± 240.6	99.8 ± 68.3	0.121
	95%-CI	17.1 - 46.7	16.3 - 102.6	11.9 - 204.9	4.2 - 374.0	47.3 - 152.3	

Data (n=9) are presented as mean ± standard deviation (m ± SD) and 95% confidence intervals (95%-CI). Δ: maximal change from baseline acutely after warm-up; CON: control warm-up; SHAM: hyperpnea at 10% maximal voluntary ventilation (MVV); WU50: hyperpnea at 50% MVV; WU80/30: hyperpnea at 80 and 30% MVV; WU70: hyperpnea at 70% MVV; FVC: forced vital capacity; FEV_1: forced expiratory volume in 1s; PEF: peak expiratory flow; $FEF_{25-75\%}$: forced expiratory flow between 25 and 75% FVC; R5: airway resistance at 5Hz; R20: airway resistance at 20Hz; R5-R20: difference in airway resistance measured at 5 and 20Hz; X5: airway reactance at 5Hz; AX: airway reactance area from 5Hz to resonance frequency; Δ: maximal change from baseline; pred: predicted; # p≤0.05 vs. CON using one-way ANOVA with repeated measures and Bonferroni post-hoc adjustments.
